# Lymelight: forecasting Lyme disease risk using web search data

**DOI:** 10.1038/s41746-020-0222-x

**Published:** 2020-02-04

**Authors:** Adam Sadilek, Yulin Hswen, Shailesh Bavadekar, Tomer Shekel, John S. Brownstein, Evgeniy Gabrilovich

**Affiliations:** 1 Google, Mountain View, CA USA; 2 Department of Social and Behavioral Sciences, Harvard T.H. Chan School of Public Health, Boston, MA USA; 3 Computational Epidemiology Lab, Boston Children’s Hospital, Boston, MA USA; 4 Department of Pediatrics, Harvard Medical School, Massachusetts, USA

**Keywords:** Infectious diseases, Epidemiology, Computational science

## Abstract

Lyme disease is the most common tick-borne disease in the Northern Hemisphere. Existing estimates of Lyme disease spread are delayed a year or more. We introduce Lymelight—a new method for monitoring the incidence of Lyme disease in real-time. We use a machine-learned classifier of web search sessions to estimate the number of individuals who search for possible Lyme disease symptoms in a given geographical area for two years, 2014 and 2015. We evaluate Lymelight using the official case count data from CDC and find a 92% correlation (*p* < 0.001) at county level. Importantly, using web search data allows us not only to assess the incidence of the disease, but also to examine the appropriateness of treatments subsequently searched for by the users. Public health implications of our work include monitoring the spread of vector-borne diseases in a timely and scalable manner, complementing existing approaches through real-time detection, which can enable more timely interventions. Our analysis of treatment searches may also help reduce misdiagnosis of the disease.

## Introduction

Lyme disease (borreliosis) is a common tick-borne illness caused by the bacterium *Borrelia burgdorferi*. It is transmitted to humans through a bite of an infected tick, and until recently was believed to affect approximately 30,000 Americans each year.^1^ A common public health approach traditionally used to count Lyme disease cases has been based on reports submitted by doctors.^1,2^ However, to gain a more comprehensive case count of Lyme disease infections, a new research investigation was recently conducted by the CDC, where researchers acquired new data from health insurance claims, clinical laboratories, and surveyed the public for self-reported Lyme disease incidents. This study led the CDC to identify that close to 300,000 Americans are affected by Lyme disease each year, making the true incidence of Lyme disease in the United States ten times higher than previously reported.^1,3^

These numbers showcase the need to develop more effective methods for monitoring the spread of the disease, which affects the health of millions of individuals in the US, since cases accumulate over time. Experts have recently begun to recognize the inaccuracy and lack of sensitivity in diagnosing Lyme disease, as well as the need to develop new strategies to measure Lyme disease.^4^

Historically, public health surveillance for Lyme disease has been limited by insufficient fidelity and lack of timeliness in reported observations. Most existing models are based on manually collected statistics that are often significantly delayed, inconsistently aggregated, and cover only a subset of jurisdictions.^1,5^ Current methods of data collection include the diagnosis of Lyme disease by physicians, confirmation of the disease by laboratory testing, manual data collection by state and local health departments, and systematic aggregation of these cases at CDC through the National Notifiable Disease Surveillance System.^6^ Although this data has been collected since 1991, many limitations in these surveillance methods make under-reporting and misclassification common in Lyme disease monitoring.^2,6^ First, health surveillance data are subject to each state’s ability to capture and classify cases, while each state has different surveillance practices and policies, which can also vary year to year depending on budgetary and personnel constraints.^2,6–8^ Second, individual states collect their data independently and asynchronously, and may close their annual surveillance dataset at different times of the year.^1^ As a result, the final case counts published by CDC do not necessarily match the annual cases reported by each state. Furthermore, final numbers are typically released two years after cases have occurred, once all the states and territories have verified their data, which limits the ability to mitigate Lyme disease in real time, especially in areas of high risk.^1^

Here we develop a complementary approach for Lyme disease monitoring, which applies supervised machine learning to highly aggregated and de-identified Google web search data. Web search has become an indispensable tool for finding health-related information. According to Pew Research, 72% of internet users say they looked online for health information in the past year, and of those 77% began their online research at a search engine.^9^ Consequently, many studies used web search data as a proxy for health concerns experienced by a population.^10–14^ Our method, called Lymelight, counts the number of users searching about the disease, and infers in which US county the disease is likely to have occurred. Lymelight starts with the absolute number of cases that it classifies as positive, and uses it to estimate the relative incidence rate for a given geographical area by dividing by the total number of users active on Google search in that area in the same time frame (2014 and 2015). Our empirical evaluation using CDC data confirms that Lymelight can accurately identify epicenters of Lyme disease and rank them in the order of significance.

In contrast to previous work,^15^ we model Lyme disease at the de-identified user level rather than query level, which allows us to estimate the number of affected individuals. Notably, our model also sheds light on the medical treatments researched by the users who have previously searched the web about Lyme disease. This data (properly aggregated and de-identified to maintain user privacy) offers public health researchers unique insights at large scale into the appropriateness of these treatments.

User-level modeling has been performed before in the context of healthcare applications. Data sources used in these studies included publicly available information such as tweets, surveys, and web postings, web search data, as well as data obtained through consented experiences (e.g., questionnaires). Coppersmith et. al.^16^ studied self-reported statements of mental health diagnosis on Twitter. Devinney et al.^17^ combined tweets and individual surveys to detect outbreaks. Sadilek et al.^14^ used de-identified web search and location data to identify foodborne illness incidents in restaurants. Paparizzos et al.^18^ used web search logs to assess individual searchers’ risk of pancreatic adenocarcinoma. Yom-Tov and Gabrilovich^19^ used sequences of individuals’ searches to discover adverse drug reactions. Youngmann and Yom-Tov^20^ combined web search and mouse tracking signals to assess people’s anxiety levels. Ben-Sasson et al.^21^ used a combination of web forum postings and screening questionnaire answers by parents to predict their child’s risk for autism spectrum disorder.

Our eventual goal is to advance the state of the art in epidemiology to a point where issues of public health significance can be quantified in a timely and actionable way using readily available online data. We call this general approach “machine-learned epidemiology”,^14^ and in this paper we report the results of applying our methodology to Lyme disease. Evaluation of Lymelight shows significant potential to improve Lyme disease monitoring methods to mitigate the spread of Lyme disease across the United States. In future research, this approach could be generalized to other vector-borne diseases, such as malaria, dengue fever, Zika fever, and Chikungunya. This becomes particularly important as climate change has the potential to affect the transmission of vector-borne diseases.^22^ We believe that methods such as Lymelight, which assess the incidence of disease in near real time, can help target and evaluate public health interventions to alleviate the negative health effects of climate change.^23^

## Results

We comprehensively evaluated the Lymelight method in several ways. In what follows, we first compare Lymelight’s county-level predictions with the official CDC statistics for the current and following years. Then, we present a quantitative analysis of relevant drug searches. In the Methods section, we also report the performance of the underlying machine-learned model for classifying individual web search queries.

### Comparing Lymelight predictions with the official CDC statistics

To evaluate the performance of our method, we computed Spearman rank correlation between the incidence rate of Lyme disease per county as estimated by our method (Lymelight) and the corresponding incidence rate from the 2015 CDC data. On the subset of counties for which de-identified search data was available, we observed the correlation coefficient of 0.92 (*p* < 0.0001, using the two-sided *t*-test to reject the null hypothesis that the two sets of data are uncorrelated), suggesting a very high degree of agreement and confirming the informative capacity of web search data.

We also evaluated the ability of our model to predict the spread of Lyme disease in the following year. Because of increased risk of exposure, the incidence of Lyme disease is known to be higher among whites,^24^ those employed in agriculture,^25^ and is related to income.^26,27^ We did not have individual-level measures on users’ demographics or socioeconomic status, and used ecological-level county variables to control for these risk factors associated with Lyme disease. Ecological proxies for individual-level measures have been validated and are often used in population health studies,^28^ especially when it is related to the context of the social and physical environment. To this end, we controlled for demographics by including the following as independent variables of the model: race, income level, and the number of people employed in forestry, agriculture, and fishing. We obtained the relevant demographics data from the United States Census Bureau’s 2011–2015 American Community Survey.^26^ In our prediction experiment, we included all the above variables alongside the Lymelight estimates for 2014, to predict CDC incidence rates for Lyme disease for 2015.

Even when controlling for demographic variables associated with greater Lyme disease risk, Lymelight estimates for 2014 was the only variable significantly associated with predicting the target variable, namely, the percentage of Lyme disease cases reported by CDC in 2015 (*p* < 0.001). Without the addition of the variable that reflects Lymelight estimates for 2014, the demographics variables only explained *R*^2^ = 15.38% of the variation of Lyme disease cases in 2015. However, the addition of Lymelight estimates for 2014 substantially increased the predictive ability of the model, allowing it to explain *R*^2^ = 78.6% of the variance, an absolute difference of 63.22%.

Furthermore, to evaluate the stability of our predictions over the years, we conducted the following two-stage experiment. In the first stage, we built a regression model that used as features the above-mentioned demographics variables together with Lymelight estimates for 2014, to predict CDC incidence numbers for 2014. Using historical data to forecast epidemiological patterns is a common methodological practice employed by the CDC. For instance, previous case reports on Ebola were used to estimate the future number of cases in the Ebola epidemic.^29^ In the case of Lyme disease, the number of cases for each year available from the CDC is relatively stable from 2014 to 2015, which enables better future estimates with the use of historical case data. Therefore, in the second stage we fixed the learned feature weights, and plugged in the Lymelight estimates for 2015, to predict CDC incidence for 2015. We observed a very low prediction error with RMSE = 0.0001571, which further confirmed the utility of the Lymelight signal.

### Using Lymelight to understand drug searches related to Lyme disease

We used Lymelight to analyze the searches for top 20 drugs by users whose searches are estimated to be positive for Lyme by our model, within a month after the first Lyme-positive query for each de-identified user. Table 1 shows the probabilities of searching for each individual drug by users who have and have not previously conducted Lyme-related web search (in what follows, we call them Lymelight-positive and Lymelight-negative cases, respectively). The purpose of this experiment was to examine treatment practices and their appropriateness. As we observe a large sample of people searching for symptoms of Lyme disease, and then searching for a variety of treatments, we can reason about the frequency of use of different treatments, as well as their suitability.

**Table 1:** Searches for drugs associated with Lyme disease sessions.

Drug searches	Lymelight-positive cases (%)	Lymelight-negative cases (%)	Chi-square	*p*-value
#1 Doxycycline^*^	26.29	0.51	2,663,557	<0.001
#2 Amoxicillin^*^	5.71	0.97	65,301	<0.001
#3 Penicillin^*^	2.56	0.53	23,698	<0.001
#4 Metronidazole^+^	2.24	0.58	16,373	<0.001
#5 Ceftriaxone^*^	2.20	0.14	70,896	<0.001
#6 Ivermectin^+^	1.94	0.18	41,860	<0.001
#7 Prednisone^#^	1.93	1.05	6057	<0.001
#8 Cefuroxime^*^	1.65	0.06	83,976	<0.001
#9 Trimethoprim/sulfamethoxazole^+^	1.56	0.58	7705	<0.001
#10 Rifampicin^+^	1.51	0.04	116,892	<0.001
#11 Clindamycin^+^	1.21	0.41	6571	<0.001
#12 Ciprofloxacin^+^	1.16	0.56	4227	<0.001
#13 Hydroxychloroquine^#^	1.06	0.12	18,039	<0.001
#14 Permethrin^+^	1.05	0.15	14,008	<0.001
#15 Clarithromycin^*^	0.97	0.07	27,183	<0.001
#16 Tinidazole^+^	0.95	0.02	87,125	<0.001
#17 Cefalexin^+^	0.94	0.41	3828	<0.001
#18 Amoxicillin/clavulanic acid^*^	0.85	0.27	4917	<0.001
#19 Fluconazole^+^	0.85	0.30	4309	<0.001
#20 Hash Oil^+^	0.83	0.21	3991	<0.001

Searches for drugs associated with Lyme disease sessions. Percentage figures show the probability of searching for the drug. The “*” symbol denotes recommended treatment for Lyme Disease (per Clinical Practice Guidelines), the “+” symbol denotes non-recommended treatment for Lyme Disease, and the “#” symbol denotes recommended treatment for arthritis.

Doxycycline was the top drug searched, and was significantly (Chi-square = 2,663,557, *p* < 0.001) more prevalent in cases identified by Lymelight as positive than in those it identified as negative. Specifically, Doxycycline had a 26% probability to be searched by a user who had previously issued queries related to Lyme disease. Amoxicillin was the second most commonly searched drug (5.71% probability, Chi-square = 65,301, *p* < 0.001), followed by penicillin (2.56% probability, Chi-square = 23,698, *p* < 0.001), with all three drugs being recommended treatments for Lyme disease.^8,9^

We found that prednisone and hydroxychloroquine, drugs for the treatment of arthritis, had a probability of 1.93 and 1.06% (respectively) to be searched in Lymelight-positive cases. Although the absolute numbers of searches for these drugs are low, their respective rankings (#7 and #13) are noteworthy. If antibiotics are not promptly used to treat Lyme disease, 60% of untreated patients develop Lyme arthritis, a late manifestation of Lyme disease that has symptoms of swelling and pain in joints similar to arthritis.^30,31^ Searches for these two drugs may be reflective of treating the symptoms rather than the underlying disease (Lyme). Alternatively, they may suggest possible misdiagnosis, as Lyme disease has overlapping symptoms with arthritis and can be misdiagnosed for it. We also found that metronidazole, trimethoprim/sulfamethoxazole, tinidazole, and fluconazole—drugs that are explicitly not recommended for the treatment of Lyme disease^32,33^—were all in the list of the top 20 drugs searched. These drugs are used to treat bacterial vaginal infections and are not effective for the treatment of Lyme disease. If Lyme disease goes untreated, emerging evidence has found an association between women with Lyme disease and higher rates of bacterial vaginal infections.^34,35^ Overall, we found that under 40% of Lymelight-positive cases searched for standard treatments recommended for Lyme disease, meaning that over 60% of these searches were outside the guidelines for the treatment of Lyme disease. Out of these 60% of drug searches, around 13% were for drugs not normally used for the treatment of Lyme disease, or drugs used to treat other conditions, suggesting a possible misdiagnosis because the underlying condition of Lyme disease may not have been correctly treated. Also, about 3% of the drug searches in Lymelight-positive cases were specifically for the treatment of arthritis, one of the most common misdiagnoses for Lyme disease. We observe that without access to electronic health record data, we were unable to determine true cases of misdiagnosis in Lymelight findings. However, the strong correlation between Lymelight output and searches for these drugs (as evidenced by high Chi-square values) may suggest a lack of treatment or delayed treatment of the underlying Lyme disease.

## Discussion

We introduced a new web-based method for real-time monitoring of the spread of Lyme disease. Our method, called Lymelight, makes its real-time predictions by leveraging web search data. To address the challenges posed by noise and ambiguity in this data, we developed a supervised machine-learned model for classifying individual queries. This model takes dozens of query-based signals as input, and estimates the probability that a query is about Lyme disease. By drawing from established clinical diagnostic criteria and using professional physician assessments, we demonstrated the accuracy of Lymelight in classifying individual queries (Fig. 2) and estimating the number of individuals with Lyme disease.

We confirmed the capacity of Lymelight to estimate the incidence of Lyme disease in counties across the United States, by showing that Lymelight predictions have 92% correlation with the official CDC data. In order to reduce the likelihood of searches from long-term sufferers, we restricted our observation period to summer months, which typically coincides with increased tick activity.^36–39^ This increases the likelihood that our model is capturing new cases of Lyme disease, and allows us to estimate the incidence thereof.

Our results show that Lymelight can estimate real-world incidence of Lyme disease much earlier and more efficiently than the official Lyme disease tracking system, which often reports data with as much as a two year delay.^3^ In the light of the recent findings that Lyme disease incidence in the United States has been considerably underreported,^32,33^ our study offers practical ways to substantially improve Lyme disease monitoring in real time.

We also showed that the output of the Lymelight model is predictive of the spread of Lyme disease in the following year. This is particularly important because warmer winters and the expansion of agricultural land development have radically increased tick populations, and consequently increased the incidence of Lyme disease.^40–45^ Although scientists are trying to build models to incorporate all the relevant parameters of the environment, these parameters fluctuate frequently across time and space,^40,43,45,46^ and their measurement requires substantial resources.^41,47–49^ This makes it difficult to predict future spread of Lyme disease, which makes the Lymelight capability to produce timely estimates of disease incidence even more important.

Prior work, notably on Google Flu trends,^50,51^ exhibited concept drift when the same model was applied over multiple years. To evaluate the concerns for the potential for drift, we calculated estimates for the entire year of 2014 and the year of 2015, which consisted of 2 years of analysis instead of 1 year that was done for Google flu trends.^52^ Several factors make Lymelight less prone to concept drift. Importantly, as we explain in the Methods section, our model is trained in a completely automated fashion, which allows us to re-train it periodically to account for possible variations in the query stream. Furthermore, based on data from the CDC, Lyme disease case counts are relatively stable over the years, making it very likely that the change in individual query classifications will not substantially affect macro-level Lymelight performance. We also note that the symptoms of Lyme disease are largely consistent from year to year, as opposed to flu that evolves over seasons^53^ and hence can result in variation in queries over time.^54^

We examined the drugs searched by de-identified users who have previously searched the web about Lyme disease (Lymelight-positive cases). Our results demonstrate that many drugs which are not recommended for Lyme disease, as well as drugs commonly associated with misdiagnosed Lyme disease, are still frequently searched by users who are also conducting Lyme-related searches. We found that doxycycline, amoxicillin, penicillin, and ceftriaxone—all drugs recommended for Lyme disease^2,8,55^—together account for less than 40% of drug searches in Lymelight-positive cases. Yet the majority of the drugs that had relatively high probability of being searched for, are suitable for conditions that are often incorrectly diagnosed instead of Lyme disease. For instance, arthritis has similar symptoms of joint pain and swelling as Lyme disease, and is often incorrectly diagnosed in patients who may in fact have Lyme disease.^32^ Two drugs typically used for treating arthritis—prednisone and hydroxychloroquine—ranked as #8 and #13 on the list of most commonly searched drugs.

Other drugs in the top 20 list included rifampicin (#10, 1.51%), which is used to treat Legionnaires’ disease,^56^ a condition that has similar muscle pain symptoms as Lyme disease;^57,58^ Clindamycin (#11, 1.21%), used for treating Babesiosis,^59,60^ another tick-borne disease;^61,62^ and ciprofloxacin (#12, 1.16%), used to treat urinary tract infections,^63,64^ a symptom that is sometimes caused by Lyme disease. All of these drugs are associated with conditions frequently diagnosed incorrectly instead of Lyme disease. The frequency of these drug searches among users who conduct Lyme-related research online, suggest that people with symptoms of Lyme disease may be misdiagnosed.

We also examined searches for drugs that are considered by the Infectious Disease Society of America as being ineffective and not recommended for the treatment of Lyme disease, because of the lack of efficacy data, absence of data, or potential harm to patients.^64^ Notably, our findings show that a large number of such drugs are still frequently searched for by users who have earlier searched about Lyme disease. For example, metronidazole (#4, 2.24%), trimethoprim/sulfamethoxazole (#9, 1.56%), tinidazole (#16, 0.95%), and fluconazole (#19, 0.85%), were all in the top 20 list.

While our empirical evaluation has shown our classifier to be fairly accurate (using data labeled by doctors according to current clinical diagnostic criteria), the classifier is not perfect. Consequently, our findings may suggest that patients searching for these drugs might have been misdiagnosed and treated incorrectly. It may also be the case that when Lyme disease goes untreated, the person develops conditions such as Lyme arthritis or bacterial vaginal infections, and subsequently searches for corresponding treatments; these searches may suggest possible lack of treatment or late treatment of Lyme disease.

Recent studies suggest that in the light of the ongoing climate change,^41^ it is imperative to collect evidence on the effectiveness of possible interventions, as well as on their implementation in the community (Type 2 and Type 3 evidence, cf.,^65^ pp. 2–3). We believe that the near real-time latency of Lymelight, paired with its ability to work at a very large scale covering entire countries, will help shed light on these very questions, and thus will help design interventions to mitigate the negative health effects of global warming.

Our approach has a number of limitations. First, and most importantly, searching for Lyme disease does not necessarily equate to a diagnosis of Lyme disease, as users might be searching for their friends and family, or even search after reading a news article about Lyme disease or about a celebrity having the disease. Thus, a Lymelight-positive case is not a confirmed case for Lyme disease. Similarly, drug searches may also not always be reflective of actual prescriptions for medications. This is an inherent ambiguity when examining Internet search patterns to better understand the incidence of any disease.

Exogenous events may also impact the statistics of Lymelight cases. For instance, media events related to Lyme disease may cause more people to search for it online. We minimized this effect by training our model to consider the plurality of one’s searches rather than isolated queries. For example, a mere query [Lyme disease] does not exceed the confidence bar for labeling the case as positive. However, if the same individual also searched for [tick rash] and [doxy], then the probability that this person investigates an active case of Lyme disease increases considerably.

Additionally, some Lyme disease sufferers might not be searching on Google. To account for that, we normalize the number of searches about Lyme disease by the total number of active users in that area (U.S. county in this study) in the same time frame, rather than the size of the entire population in the area. While Lymelight by design doesn’t explicitly distinguish between searching on behalf of oneself or someone else (e.g., a family member), this distinction largely disappears in aggregation. Whether a user is researching their own infection or on behalf of someone else, that positive instance will increment the estimated case count in the appropriate geographical area and time. There may be, however, instances where a single user researches multiple ongoing cases of Lyme. In these rare cases, the incidence rate would be slightly underestimated.

An important limitation of our approach is the scarcity of Lyme-related search data in many counties, which precludes us from computing Lymelight results there in order to maintain strict privacy of our users. We discuss this limitation in detail in the section “Lymelight validation”, and show the properties of the counties for which we can and cannot compute results. This limitation is substantially alleviated for more common diseases, such as foodborne illness, which we have studied using web search data in our prior work.^14^ In our future work, we plan to address this limitation by applying machine-learned epidemiology to additional, more common diseases, as well as by experimenting with higher levels of aggregation.

Research has shown that the risk of Lyme disease increases with forest fragmentation, which is attributed to houses with larger lot sizes.^66–68^ Such houses can be associated with socioeconomic groups with higher income, whose members are able to afford larger land lots for dwelling, making the population at risk include those with higher socioeconomic status. However, it has been noted that Lyme disease is often under-reported in rural areas as well as in areas with lower socioeconomic status, because of the lower presence of and access to medical care facilities there. Therefore, the population at risk might also not be fully encompassed within this study. Yet, although these areas may have lower Internet access, which may limit detection of the disease cases using online signals, individuals in these areas may be more reliant on the Internet for information about their disease. Consequently, Lymelight has the potential to improve detection of Lyme disease throughout the country, including in areas with lower socioeconomic status.

Another limitation of our study is the lack of access to electronic health records (EHR). To protect privacy, it is by design impossible to link Lymelight inferences with EHRs. As a result, without EHR data, we are unable to confirm Lymelight-positive cases, nor are we able to determine cases of possible misdiagnosis. If a Lymelight-positive case searched for the correct drug, we assumed that a correct diagnosis of Lyme disease was given, but cannot confirm this is truly a case of Lyme disease. Similarly, if a Lymelight-positive case searched for a drug not recommended for Lyme disease or recommended for a disease that is often misdiagnosed for Lyme disease such as arthritis, we again, can only assume this is a misdiagnosis. Without ground truth data from EHRs, we are unable to estimate the true disease incidence. However, the high correlation at an aggregate population level we observed between Lymelight and CDC data suggests strong evidence supporting that Lymelight accurately estimates the cases of Lyme disease.

Symptoms of Lyme disease can also be long-term,^32^ thereby making it difficult to determine if the cases that Lymelight classifies as positive are actually new cases of Lyme disease. According to CDC, 10–20% of patients who undergo full course of antibiotics have “post treatment Lyme disease syndrome”,^59,69^ which manifests as lingering fatigue, pain, or joint and muscle aches.^70–73^ Furthermore, although an early course of antibiotics can usually effectively treat Lyme disease, diagnosis and treatment can often be delayed, resulting in long-term symptoms of headaches, chronic gastrointestinal problems, memory loss, stiffness of joints, and speech impairment.^74^ Without access to electronic health records (EHR), we are unable to confidently determine if a Lymelight-positive case is acute or chronic. However, we trained our model based on the CDC definition of a confirmed incident case, which is defined as having evidence of infection defined by clinical presentation of symptoms and likelihood of exposure to ticks. Although chronic Lyme disease patients have persistent symptoms of Lyme disease, these patients would likely have previously received a diagnosis because of their signs of tick exposure, which would not be present for chronic Lyme disease. Similarly, we trained Lymelight using queries based on symptoms of Lyme disease and the likelihood of exposure to ticks, this making Lymelight estimate incidence and not prevalence.

There could be additional reasons why the correlation between Lymelight predictions and CDC data is imperfect, suggesting possible limitations of the traditional surveillance mechanisms. Some patients may not be diagnosed if they do not seek medical care, or may be misdiagnosed (as suggested by our analysis of drug searches), and so they will not be included in the official statistics. Other cases might not be timely reported in the correct observation period.

Digital methods such as Lymelight are not intended to replace traditional epidemiological methods but are instead leveraging online data to mitigate some of the existing gaps. Findings from this study can offer greater sensitivity and speed of disease detection. Future work should explore how digital monitoring methods can be employed to enhance current epidemiological practices, through studies that can further validate the capacity of our machine-learned infrastructure to identify cases of misdiagnoses and under-reporting.

Although traditional methods of capturing infectious disease epidemics such as Lyme disease have begun to incorporate new data collection methods and streams of information, current public health approaches to epidemiological surveillance have a number of limitations. For instance, the accuracy of the data aggregated by CDC is subject to individual states’ abilities to capture and classify cases in a timely manner, which depend on states’ budget, personnel, and strategies, which vary not only between states but also from year to year.^2,6,75,76^ Individual states have their own dataset preparation timeline compared to that of the CDC, and states may close their annual surveillance period at a different time than the CDC, making the final case counts published by the CDC not reflective of the actual numbers published by each state agency.^2,6,75,76^ Finally, current data collection practices usually record statistics by the county of residence and not the county of exposure.

These limitations of traditional surveillance tools often result in a lack of consistency in reporting, and changes in reported cases from a state do not always represent a true change in disease incidence.^2,6,7,76^ Additionally, traditional data collection routines are highly labor intensive and resource-heavy, which may result in under-reporting and misclassification of cases, because not every case of Lyme disease is reported to the CDC and in some instances cases that are reported as Lyme disease are due to another cause.^2,6,76^

Lymelight may also help capture additional cases not traditionally captured by counting visits to hospitals and doctor’s offices, and thus can help reduce under-reporting that has been often seen with vector-borne diseases. While it has been noted that areas with lower socioeconomic status have less Internet access, these areas also have lower access to health care facilities, and hence have fewer resources to diagnose, detect and capture disease cases. Thus, even with limited Internet access (e.g., in public libraries), individuals living in these areas may be more reliant on the Internet for information on their disease prior to visiting a healthcare facility, or using web searches as a primary source to self-treat themselves at home. Therefore, new digital approaches should be deployed in lower resourced and rural areas to evaluate their potential for identifying cases within these areas that traditional sources have had difficulty capturing.

Modeling of Lyme disease based on web-search data offers a complementary approach to traditional methods, and can mitigate the above mentioned limitations. Our results showed that Lymelight was able to adequately estimate Lyme disease incidence compared to the official Lyme disease statistics published by the CDC. Unlike the traditional methods, Lymelight retrieves data through de-identified online search queries, and has the potential to assess the disease incidence without the additional resource cost incurred by traditional epidemiological methods. Since Lymelight can use data across the entire country, a more accurate and consistent estimation of the disease incidence can be produced.

Future studies should explore approaches that combine the benefits of traditional disease tracking mechanisms with those offered by Lymelight.

The most significant benefit of using the proposed monitoring infrastructure is the potential for faster and earlier detection of the spread of disease. Notifications by traditional surveillance systems are highly dependent on reports by doctors or laboratories.^76^ However, evidence has indicated that individuals often search for health information online, especially at earlier stages of their illness, before making a medical visit; they also sometimes use the web to decide whether to visit a doctor.^54^ Therefore, by utilizing de-identified web search queries there is a potential to provide earlier signals of disease detection than clinical or laboratory reporting. Research has also shown that some individuals choose to search for health information online instead of making a medical visit.^54^ Therefore, not only can search queries help detect cases earlier, but they may also help capture cases not traditionally reported, which can help reduce under-reporting.

Our present study, by design, makes it impossible to link web search data with data from electronic health records. However, we recognize that such data could make our findings more comprehensive. Future studies could consider ways to validate online signals using data from electronic health records, in a privacy-preserving way, to better estimate disease incidence, as well as identify possible cases of misdiagnosis.

Aggregated statistics from searches for drug treatments may also be useful for identifying incorrect treatment practices. Future work may also combine information about drug-related searches with diagnostics from laboratories, in order to identify areas with higher risk for miscommunication, misclassification, or poor reporting procedures. Through this information, digital data could be used to identify areas in need of better provider training or resource management. An additional future direction could quantify distributions over the time elapsed between the first potential evidence of Lyme and subsequent searches for various drugs. More broadly, digital data can be a low-cost complement to traditional disease tracking, and future work should involve coordination between traditional and digital methods to help mitigate the gaps.

## Methods

### Machine-learned query classification model for Lyme disease

In order to estimate county-level incidence rate of Lyme disease from Google search traffic, we first need a scalable way to estimate which queries are about the disease. Using this core module, Lymelight calculates the proportion of de-identified users who are searching for Lyme disease. Our model aggregates the statistics at county level, thereby preserving privacy and allowing direct comparison with the official CDC data. The remainder of this section explains each step of this process in detail.

Lymelight leverages as input search sessions from de-identified signed-in users, thereby eliminating the need to depend on less reliable mechanisms of deduplication (e.g., using Internet cookies or IP addresses), particularly over multiple days of data. This is important in the case of Lyme as it involves long incubation periods of up to 30 days, and even longer disease progression. User data has been de-identified to maintain user privacy.

The key challenge here is the inherent noise and ambiguity of individual search queries. For example, the query [*tick bute*] is predictive of Lyme disease incidence rate, but also contains a typo and does not convey information about the details of the tick bite (e.g., whether the bite site got swollen or itchy). We solve this challenge by developing a privacy-preserving supervised machine-learned classifier of Lyme disease queries, which mitigates this noise by leveraging a collection of signals beyond the query string itself. To this end, we use aggregated search results for the query and aggregated clicks on those results.

Web search queries and online data have been found useful in prior public health research.^10–12,77^ Most relevant to this paper is Google Flu Trends,^13,78^ which tracked the proportion of 45 specific whitelisted flu-related queries among all the queries from a given geographical region. The selection of these whitelisted queries was not machine learned, and therefore was more susceptible to topic drift and noise over time. In contrast, Lymelight uses automated learning techniques to identify the infinite variety of ways in which symptoms, treatments, and other aspects of Lyme disease can be described in natural language. Furthermore, Lymelight improves the understanding of individual queries, which are usually short and ambiguous, using the search results returned for them.^79^ As a result, Lymelight can be continuously re-trained and therefore adapted to changes in the query stream and web use.

Finally, the Google Flu Trends study estimated the general query volume about the disease, rather than the actual incidence rate as we do herein. This distinction is important for two reasons. First, certain web users, such as medical professionals or academic researchers, may issue a significant number of relevant queries, which does not necessarily imply higher incidence of the disease in the population. We believe that using a machine-learned classifier at the user level allows us to estimate the true incidence rates of the disease in a more robust and accurate way. Second, working at the user level allows us to conduct novel kinds of analysis, such as studying the treatments searched by users who had previously searched about Lyme disease. This analysis, which allows us to reason about the appropriateness of those treatments for Lyme disease, was made possible thanks to our user-level modeling.

Other publicly available systems that aggregate online signals, such as Google Trends and Yahoo! Buzz, have also been used in epidemiology research. Google trends allows its users to examine the popularity of top web search queries, with the ability to focus on different geographical areas and time periods. It has been widely used in prior published work on a variety of diseases,^65,80–84^ including Lyme disease.^85,86^ Our proposed approach expands the capabilities of Google Trends and features several types of functionality that are particularly important in epidemiological research and applications. Specifically, our method counts affected individuals rather than queries, which allows us to assess disease incidence more reliably. Whereas Google Trends is by design limited to the most frequent (so-called “head”) queries, Lymelight classifies all queries in the query logs, including the less frequent ones (“torso” and “tail” queries, which cumulatively account for a non-negligible part of the overall query volume). Furthermore, by classifying all queries we gain generalization ability, and thus can account for misspellings, syntactic variants, as well as semantically related queries. In contrast, when using Google Trends researchers have to decide in advance on the list of queries to focus on, because they need to explicitly submit every query to the Trends engine to get its occurrence statistics. Finally, whereas Google Trends data is only offered at the level of entire states and a few large metro areas, Lymelight can examine data at the finer spatial granularity of counties, while still aggregating data and maintaining high standards of privacy.

Yahoo! Buzz was a conceptually similar tool offered by the Yahoo! Search engine (the tool has since been discontinued), which was used to study search activity related to the 23 most common cancers in the United States^87^ and other studies. A survey of similar tools that have been used in epidemiology research can be found in.^88^

### Data description

We applied our query classification model to the aggregation of all English-language web search queries from the United States spanning years 2014 and 2015, and estimated the proportion of user sessions that suggest significant evidence of online research about Lyme disease. The query-level confidence threshold was chosen at 93%, which is the optimal operating point established in our empirical evaluation (see below). We filtered out users who are unlikely to be investigating an active case of Lyme disease but are still querying for it, e.g., those who may be researching the disease for academic purposes, searching for symptoms of a family member or a friend, or searching about a news story related to Lyme disease. To do so, we only counted users who issued three or more queries that our model identified as Lyme-related.

This processing has been performed on data from logged-in users who opted to record their web search history. At the beginning of processing, queries have been de-identified. This allowed Lymelight to count the number of users who have issued queries about Lyme disease, and those who later issued queries about relevant drugs, in a privacy-preserving way. All the processing has been done automatically, including the labeling of training examples for query classification (both positive and negative examples), so that no training example query was analyzed by humans.

This work has been performed in accordance with relevant guidelines and regulations, and approved by Google. The data has been collected with users’ consent in accordance with the Google Terms of Service and Privacy Policy. This study was designated as non-human subjects research by Boston Children’s Hospital Institutional Review Board (IRB).

### Query-level classification model

We built a log-linear maximum entropy model^89^ that estimates for a de-identified search query the probability that the query is about Lyme disease. Model training happens in a supervised way from automatically inferred labels. This allows us to deploy the model at scale and avoid relying on human raters, which can be very costly, and also maintains user privacy, as no training query is looked at by humans.^14^ To achieve this aim, we observe that queries that lead to significant time spent on web pages about Lyme disease (broadly defined, including pertinent treatments and symptoms) are more likely to be about Lyme disease. Examples of such pages are the Wikipedia article about Lyme disease or the CDC web site devoted to the disease (https://www.cdc.gov/lyme/index.html).

Anchoring on web pages allows us to regularize over the noise in individual queries, which—unlike pages—tend to be short, ambiguous, and often ungrammatical. Our training pipeline automatically aggregates queries leading to these websites, and uses them as positive examples. Then, it randomly samples other queries (in proportion to their frequency in the overall query stream) to serve as negative examples. The Lymelight model is trained using these two automatically-labeled sets of queries. The resulting model estimates the probability that a query is used for online research about Lyme disease (producing a score between 0 and 1 for each query), and does not require any human effort or manual inspection of individual queries.

The model has a feature space of dimensionality 50,000 and uses feature hashing for compactness.^90^ The features consist of unigrams and bigrams extracted from the query string, as well as from the search result URLs, snippets (short summaries of each result displayed by the search engine), and web page titles. We also construct features based on Google’s Knowledge Graph^91^ annotations of the concepts mentioned in the query.

### Feature analysis

To explore the patterns that our model has automatically learned from the training data, we examine the top 50 n-gram features ranked by information gain (Table 2).^92^ We computed information gain using the automatic labels, which were obtained as explained in the previous section.

**Table 2:** Top 50 classifier features, ranked by information gain.

Feature	Information gain (in bits of information)
Lyme	1.10E−03
Lyme disease	1.08E−03
Tick	6.90E−04
Ticks	6.60E−04
Of lyme	6.40E−04
Disease	6.20E−04
[Lyme disease] (KG concept)	5.50E−04
A tick	5.10E−04
[Tick] (KG concept)	4.70E−04
Parasites	4.50E−04
Tick borne	4.40E−04
Tick bite	4.30E−04
Tick bites	3.80E−04
[Pathogenic bacteria] (KG concept)	3.80E-04
Borrelia	3.70E-04
For lyme	3.50E-04
Conditions lyme	3.50E-04
Diseases	3.40E-04
Bite	3.30E-04
Borne	3.30E-04
Burgdorferi	3.30E-04
cdc	3.30E-04
[Disease vectors] (KG concept)	3.20E-04
[Disease] (KG concept)	3.20E−04
Borrelia burgdorferi	3.20E−04
Disease cdc	3.20E−04
Disease is	3.10E−04
[Infectious diseases] (KG concept)	3.10E−04
Ticks are	3.10E−04
Ticks and	2.80E−04
The tick	2.80E−04
[Disease or medical conditions] (KG concept)	2.80E−04
Symptoms	2.70E−04
Blacklegged	2.60E−04
Of ticks	2.50E−04
Disease symptoms	2.50E−04
The bite	2.40E−04
Of tick	2.40E−04
Disease lyme	2.40E−04
Lyme disease	2.40E−04
Health	2.30E−04
Infection	2.20E−04
Bites	2.20E−04
Treatment	2.20E−04
Infected	2.20E−04
Rash	2.20E−04
Transmitted	2.20E−04
About lyme	2.20E−04
With lyme	2.10E−04
Deer ticks	2.00E−04

Top 50 features, ranked by information gain. KG concepts are those found in the Google Knowledge Graph.

We see that most features are strongly related to Lyme disease (e.g., “borrelia”) and ticks (e.g., “blacklegged”). Some features are broad categorical terms (e.g., the Knowledge Graph concept of pathogenic bacteria), which enables the model to learn a good decision boundary between positive and negative Lyme cases.

The features we used include plain text unigrams and bigrams (e.g., “tick”, “lyme disease”), as well as more general concepts such as those found in the Google Knowledge Graph (e.g., “Lyme disease” or “Parasites”). Occasionally, query terms are misspelled, yet if they appear often enough they can still be informative as features (e.g., “Lymedisease” in one word). We did not perform stemming and did not remove stop words.

### Evaluation of the query-level classification model

We evaluated our model on two levels. In this section, we discuss its performance at the micro level of de-identified individual queries, and we compare the output of the Lymelight query classification to the human-provided labels. In the next section, we discuss the macro level performance of Lymelight, where we compare the incidence it computes for US counties with that available from the CDC.

Lymelight relies on the ability to determine if a web search query is about Lyme disease. We evaluated this ability on a set of 5000 queries, and collected a total of 50,000 expert judgements. To evaluate the precision and recall of our query classification model, we employed two types of human judges: non-medical professionals as well as licensed medical doctors (MDs), trained in various medical specialties and located across the United States. Experts in both rater groups were unknown to and independent of the authors. Additionally, the raters were not aware of this research and did not know the purpose of the task. They were engaged by a third party provider—also independent of the authors—that ensured proper qualifications of the raters.

**Fig. 1:**
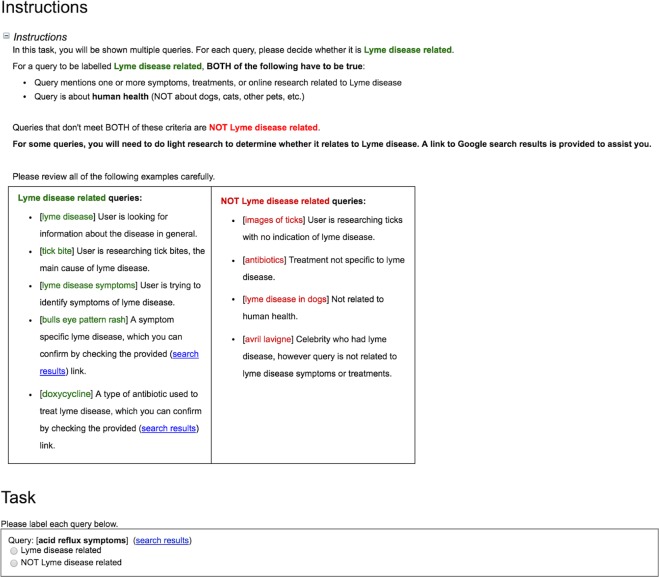
Task definition for obtaining human judgements on queries. The same template was used to solicit labels from non-medical professionals as well as from medical doctors.

The raters assessed the search queries to identify if a query was related to Lyme disease based on current clinical diagnostic criteria. The raters were presented with the task shown in Fig. 1.

Five non-medical professionals and five MDs independently judged the relevance of each query to Lyme disease. Inter-rater agreement—measured by Krippendorff’s alpha^68^—was 0.7 over all judgements collected from both groups, indicating a high agreement. We evaluated the Lymelight model by aggregating all ratings from the ten raters (five MDs and five non-professionals) for each query using majority-vote rule. Ties were broken using majority rule over MD votes. Since searches potentially related to Lyme disease are relatively rare, we designed a high-recall filter that leverages clicks on web pages about Lyme (annotated with Knowledge Graph topics described at https://www.google.com/intl/en_us/insidesearch/features/search/knowledge.html). Specifically, we collected a large set of queries that led to clicks on such topical web pages, and then sampled queries out of this set according to their traffic weight. All queries were de-identified and highly aggregated to preserve privacy.

Of the resulting 5000 queries, 9% (450 queries) were labeled by human annotators as positive for Lyme disease, and the rest were labeled as negative.

Overall query-level agreement across annotators was 80%, with MDs being more conservative (saying “no Lyme disease” with 15% higher probability). Most of the disagreements between the two rater groups occurred for queries that were ambiguous (e.g., had a 3:2 vote split inside each rater group). Disagreement on queries that were less ambiguous was only 8%.

**Fig. 2:**
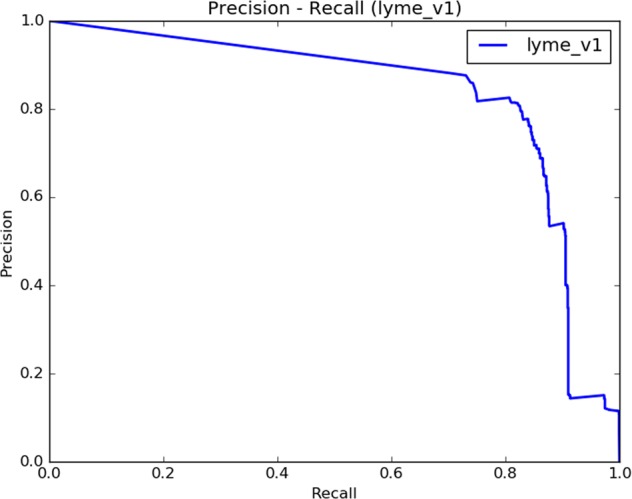
Precision-recall plot for the Lymelight query classification model.

We used the query dataset labeled as explained above to evaluate our query classifier (Fig. 2). In addition to the area under the receiver operating characteristic (AU-ROC), we examined the area under the precision-recall curve (AU-PR, cf. Fig. 2) because we face a class-imbalance problem where the number of estimated negative queries far exceeds the number of positives. Since AU-PR incorporates the prior probability of a class, it provides a better estimate of real-world performance in the presence of class imbalance. We found the query classifier to exhibit robust performance over a range of operating points, with AU-ROC = 0.99 and AU-PR = 0.83. At the optimal decision point, we observed Precision = 0.81, Recall = 0.82, and *F*1 = 0.82 (the *F*1 score is a harmonic mean of precision and recall^69,70^).

### Lymelight validation

In the previous section, we presented the results of Lymelight classification accuracy on de-identified individual queries. Here we discuss its performance on predicting Lyme disease incidence at the level of US counties.

We validated Lymelight’s capacity to estimate the true incidence of Lyme disease using a two-pronged approach. First, we compared Lymelight predictions with the incidence of Lyme disease as reported by CDC at the US county level for the same year. Second, we showed that Lymelight estimates can also be used to better predict Lyme disease incidence at the same location in the following year.

The Lyme disease case counts prepared by the CDC are first individually collected and verified by state and local health departments (all personally identifiable information is removed during the collection phase prior to reporting to CDC). CDC makes this data publicly available approximately two years after the cases were originally recorded. At the time of our experiments (August 2017), the most current publicly available dataset with CDC Lyme statistics dated back to 2015. Therefore, we compared the incidence of Lyme disease in 2015 as reported by the CDC to that predicted by Lymelight using web search logs from the same year.

The percentage of Lyme disease cases in the population was calculated by dividing the number of reported cases of Lyme disease in CDC data, by the total population of each county retrieved from the United States Census Bureau’s 2011–2015 American Community Survey.^26^ Lymelight data was normalized in a similar way, by dividing the number of users estimated to research Lyme disease online by the total number of active users in the same county and the same time frame. The evaluation data was restricted to summer months within each year (June to August), as this is the most active season for ticks and has the greatest incidence of new Lyme disease cases.^69^ We computed Spearman rank correlation at the county level between the CDC data and Lymelight predictions, in order to assess the accuracy of our model in identifying the incidence of Lyme disease.

CDC incidence data is indexed by the county of residence of the patients. We use coarse query location aggregated at the US county level to match existing CDC datasets and to facilitate paired evaluation on the same counties. Each Lymelight case represents a web search user detected by our model as conducting online research about Lyme disease; it is assigned to a county by taking a majority vote among the locations of all the user’s queries. The county was inferred using coarse IP address-based geocoding in accordance with the Google Terms of Service (https://www.google.com/policies/terms/). We ensured user privacy by using aggregation buckets at county level with at least 50 data points in each bucket, and by automatically removing all potentially personally identifiable information from the queries to de-identify the data.

In order to preserve the privacy of our users, we required that there should be at least a certain number of users in each bucket that satisfied all the selection criteria, namely, a minimum number of unique users who issued web search queries during the analysis period (summer months of 2015), and also specifically issued at least three queries that Lymelight classified as positive. This privacy aggregation threshold restricted Lymelight results to only a subset of counties. More specifically, Lymelight results are not available for two classes of counties: those with small populations, where the number of searchers is small to start with, and those with low endemic rates of Lyme disease, because the number of users searching about Lyme is small.

Under these privacy-preserving constraints, Lymelight produced results for 33 counties, which offer a small but diverse sample of all US counties. In what follows, we first analyze the properties of this sample (which we henceforth refer to as LL2015), and then compare the Lymelight ranking of counties to that produced by the CDC.

**Fig. 3:**
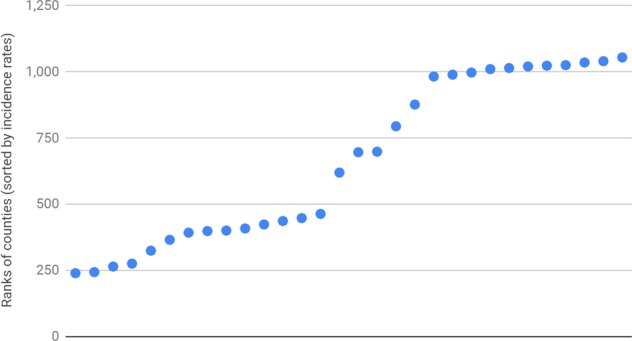
Rank coverage by LL2015 counties. The plot shows ranks at which LL2015 counties appear in the list of all counties, which is sorted by decreasing order of incidence rates according to CDC. We observe near-uniform coverage between ranks 240 and 1055.

We sorted all the 3193 counties for which CDC data was available in 2015, in decreasing order of incidence rates. Figure 3 depicts the ranks at which LL2015 counties appear on this list. Our list effectively samples counties on the CDC list at ranks 240–1055. Below rank 240, we have counties with the highest rates of Lyme disease - those happen to be located primarily in the northeast, and are of smaller size, so they do not have enough searchers to meet the aggregation threshold. We have a near uniform sample of counties at ranks 240–1055. Towards the end of this list are large counties such as Santa Clara County, CA, or Los Angeles County, CA—they have low rates of Lyme disease, but have huge populations that allow them to meet the aggregation threshold. Above rank 1061, counties have zero rates of Lyme according to CDC data, which explains the scarcity of Lyme-related searches there.

We also computed the total number of cases, which according to CDC happened within the LL2015 subset of counties in 2015. These cases summed up to 5482, or 14.4% of the total burden of 38,069 cases in the US in 2015 according to CDC. The total population of these counties amounted to 63.4 million people, or 19.7% of the total US population in 2015.

Table 3 shows the ranking of LL2015 counties according to CDC and according to Lymelight, when sorted in decreasing order of incidence rates computed for each data source.

**Table 3:** Ranking of counties according to CDC and according to Lymelight.

Rank	CDC	Lymelight
1	New Haven County, Connecticut	Fairfield County, Connecticut
2	Montgomery County, Pennsylvania	New Haven County, Connecticut
3	Chester County, Pennsylvania	Chester County, Pennsylvania
4	Fairfield County, Connecticut	Suffolk County, New York
5	Middlesex County, Massachusetts	Middlesex County, Massachusetts
6	Essex County, Massachusetts	Allegheny County, Pennsylvania
7	Hartford County, Connecticut	Essex County, Massachusetts
8	Montgomery County, Maryland	Westchester County, New York
9	New York County, New York	Hartford County, Connecticut
10	Suffolk County, New York	Montgomery County, Pennsylvania
11	Hennepin County, Minnesota	Suffolk County, Massachusetts
12	Fairfax County, Virginia	Fairfax County, Virginia
13	Westchester County, New York	Hennepin County, Minnesota
14	Allegheny County, Pennsylvania	Montgomery County, Maryland
15	Suffolk County, Massachusetts	New York County, New York
16	Kings County, New York	Philadelphia County, Pennsylvania
17	Philadelphia County, Pennsylvania	Nassau County, New York
18	Queens County, New York	Wake County, North Carolina
19	Nassau County, New York	Kings County, New York
20	DuPage County, Illinois	DuPage County, Illinois
21	Wake County, North Carolina	Oakland County, Michigan
22	Cook County, Illinois	Queens County, New York
23	Orange County, Florida	Cook County, Illinois
24	Santa Clara County, California	Santa Clara County, California
25	Broward County, Florida	King County, Washington
26	Oakland County, Michigan	San Diego County, California
27	Miami-Dade County, Florida	Orange County, Florida
28	Travis County, Texas	Travis County, Texas
29	King County, Washington	Miami-Dade County, Florida
30	San Diego County, California	Los Angeles County, California
31	Harris County, Texas	Tarrant County, Texas
32	Tarrant County, Texas	Broward County, Florida
33	Los Angeles County, California	Harris County, Texas

Ordering of LL2015 counties according to CDC and according to Lymelight, in decreasing order of incidence rate computed by each source.

### Drug searches

Lyme disease may often be misdiagnosed,^93^ resulting in inappropriate treatments. To this end, we investigated whether users who search for Lyme disease would subsequently search for clinically recommended treatments for this disease. We made an assumption that people who search for specific prescription drugs are likely to have been prescribed and are taking the drugs. Therefore, to identify the drugs that are being prescribed for Lyme disease, we calculated the probability of a drug to be searched in cases identified by Lymelight as positive and negative. Then, we used Chi-square test to determine if a drug was searched for significantly more frequently in the Lyme-positive cases. As in all other experiments, we ensured user privacy by de-identifying the queries and by automatically removing all potentially personally identifiable information from the queries.

We performed this analysis on a list of recommended and non-recommended treatments for Lyme disease, which we compiled using the Clinical Practice Guidelines for the treatment for Lyme disease.^7,94–96^ We specifically included in our list those drugs that are often prescribed for conditions whose symptoms overlap with those of Lyme disease, such as arthritis, babesiosis, and urinary tract infections. This allowed us to analyze if users who had searched for Lyme disease were subsequently searching for inappropriate treatments.

### Reporting summary

Further information on research design is available in the Nature Research Reporting Summary linked to this article.

## Supplementary information

Reporting Summary

## Data Availability

The data that support the findings of this study were obtained from Google, Inc. under a license for the current study. Data may be available from the authors upon reasonable request and with permission of Google, Inc.

## Code availability

The log-linear maximum entropy classification model used in this study was implemented using a regression tool substantially similar to that in TensorFlow and the text preprocessing tools substantially similar to the Natural Language Toolkit. Statistical analysis was performed using R software.
